# Arbitrarily polarized bound states in the continuum with twisted photonic crystal slabs

**DOI:** 10.1038/s41377-023-01090-w

**Published:** 2023-03-07

**Authors:** Haoye Qin, Zengping Su, Mengqi Liu, Yixuan Zeng, Man-Chung Tang, Mengyao Li, Yuzhi Shi, Wei Huang, Cheng-Wei Qiu, Qinghua Song

**Affiliations:** 1 Tsinghua Shenzhen International Graduate School, Tsinghua University, Shenzhen, 518055 China; 2 Department of Electrical and Computer Engineering, National University of Singapore, Singapore, 117583 Singapore; 3 Institute of Precision Optical Engineering, School of Physics Science and Engineering, Tongji University, Shanghai, 200092 China; 4 Key Laboratory of Multifunctional Nanomaterials and Smart Systems, Suzhou institute of Nano-Tech and Nano-Bionics (SINANO), Chinese Academy of Sciences (CAS), Suzhou, 215123 China

**Keywords:** Metamaterials, Photonic crystals

## Abstract

Arbitrary polarized vortex beam induced by polarization singularity offers a new platform for both classical optics and quantum entanglement applications. Bound states in the continuum (BICs) have been demonstrated to be associated with topological charge and vortex polarization singularities in momentum space. For conventional symmetric photonic crystal slabs (PhCSs), BIC is enclosed by linearly polarized far fields with winding angle of 2*π*, which is unfavorable for high-capacity and multi-functionality integration-optics applications. Here, we show that by breaking *σ*_*z*_-symmetry of the PhCS, asymmetry in upward and downward directions and arbitrarily polarized BIC can be realized with a bilayer-twisted PhCS. It exhibits elliptical polarization states with constant ellipticity angle at every point in momentum space within the vicinity of BIC. The topological nature of BIC reflects on the orientation angle of polarization state, with a topological charge of 1 for any value of ellipticity angle. Full coverage of Poincaré sphere (i.e., $- {{\pi \over 4}} \le \chi \le {{\pi \over 4}}$ and $- {{\pi \over 2}} \le \psi \le {{\pi \over 2}}$) and higher-order Poincaré sphere can be realized by tailoring the twist angles. Our findings may open up new avenues for applications in structured light, quantum optics, and twistronics for photons.

## Abstract (web summary)

Bilayer twisted photonic crystal slabs enable arbitrarily polarized bound states in continuum (BICs) covering full Poincare sphere.

## Graphical abstract

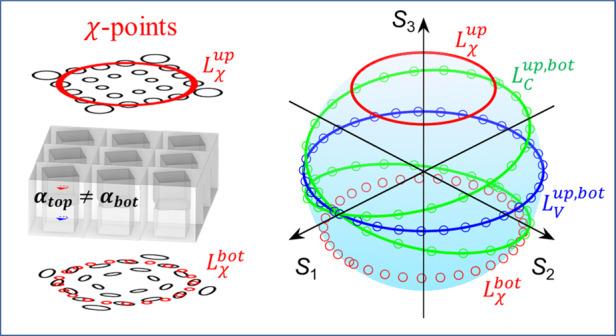

## Introduction

Photonic crystal slab (PhCS) is dielectric structure with periodically modulated refractive index and finite thickness, supporting Bloch modes that are mostly leaky with finite lifetimes^1^. Destructive interference or symmetry mismatch can make these modes become nonradiative with infinite lifetime, that is, bound states in the continuum (BICs)^2–7^. The mode at BIC remains localized and cannot couple with free-space radiations even in a continuous spectrum carrying energy away. Since there is no outgoing power and thus undefined polarization vector, BIC manifests as a momentum space singular point representing vortex center (V point) in the far-filed polarization state^5,8–10^. Conventionally, when the PhCS supporting BIC maintains *σ*_*z*_-symmetry (up-down mirror symmetry) and in-plane inversion symmetry, the far-field is linearly polarized and the polarization angle winds around the V point, generating topological radiation pattern quantized by topological charges^5,10–12^. These winding topologies of resonances in momentum space can inherently act like spatially winding configurations in real space for generating optical vortex beams^13–16^. The topological property guarantees the robustness of BIC against change of system’s parameters. Recently, modulating polarization of light and exploring polarization singularities using PhCS has been of greet interest due to abundant polarization features emerging in momentum space^17–20^. To overcome the limit of only linearly polarized resonance around BIC, the in-plane inversion symmetry of square PhCS is broken into trapezoid and triangle, which causes the V point at BIC decomposed into two circular polarized states (C points) and equator lines on the Poincaré sphere (L lines)^10,21^. This broken symmetry enriches the features of polarization modulation using BIC topology and achieves full coverage of Poincaré sphere. Later, with a honeycomb lattice, it has been demonstrated that C points can occur near BICs and Dirac points with preserved in-plane inversion symmetry, which remarkably contributes to diversity of polarization around BIC^22^. While for PhCS with inversion symmetry but broken *σ*_*z*_-symmetry, two C points emerge without splitting the V point, and the evolution demonstrates merging processes governed by the global charge conservation^23^. These findings promote the applications of PhCS supporting BIC for polarization modulation and manipulating various polarization singularities in momentum space.

However, in previous studies, the circularly polarized states spawning from BIC can only occur in pairs at off-Γ point due to the conservation of an integer topological charge induced by the V point, and pure elliptical/circular polarization states within a large span of momentum space cannot be achieved^10,23^. The relevant circular and elliptical polarization states originate from destroyed radiative modes that are almost linearly polarized enclosing BIC. This is due to unperturbed *σ*_*z*_-symmetry and unidirectional scattering of eigenmode of the PhCS^24–26^. Although broken *σ*_*z*_-symmetry has been analyzed in ref. ^23^ in the one-dimensional PhCS, the lack of rotation asymmetry still ends up with unidirectional guided resonances with global charge of zero. Most recently, the concept of twistronics has been extended from two-dimensional heterostructures to optics like twist-stacked metamaterials and chiral PhCS, which provide new platforms for tailoring chiral-optical effects and elliptical polarizations with new degree of freedom^27–32^.

In this letter, based on the ellipticity angle *χ* of polarization states, we define the polarization singularity induced by the Γ-BIC from the twisted PhCS as *χ* point, which can be encircled with constant ellipticity angle *χ*. BIC in the twisted PhCS has infinite quality factor robust against rotation angle of the hole and is encircled by far-field polarization state carrying the same ellipticity angle (*χ*). Taking advantage of BIC-induced topological distribution of orientation angle (*ψ*), polarization states around BIC encircles a full latitude of the Poincaré sphere for each BIC. The ellipticity angle of *χ* point can be arbitrarily controlled by changing the rotation angle of the hole, resulting in a full coverage of Poincaré sphere of the radiating polarization states.

## Results

### Concept of constantly distributed and arbitrary polarized BIC

**Fig. 1:**
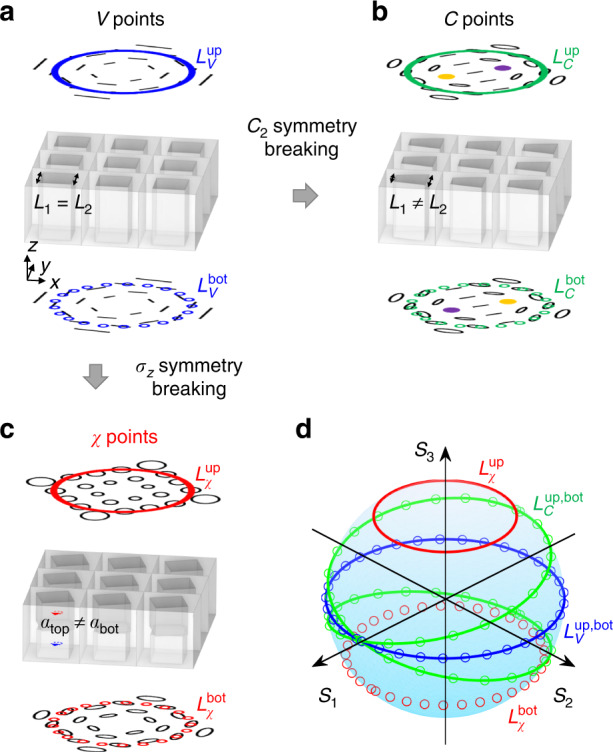
Conventional *σ*_z_-symmetric and twisted photonic crystal slab (PhCS) supporting BICs at the Γ point. **a** Schematic of a conventional PhCS with etched square-hole standing in free space and its far-field polarization states distribution in momentum space near the V point at BIC. **b** Breaking in-plane inversion symmetry of the PhCS results in elimination of BIC and two C points spawning from the V point. The radiation in upward and downward remains the same. **c** The twisted PhCS by rotating the top hole with an angle of *α*_*top*_. Breaking *σ*_*z*_-symmetry results in asymmetric radiation of elliptical polarizations surrounding BIC upward and downward. The BIC is redefined as a *χ* point referring to arbitrary polarization states with the same ellipticity *χ* enclosing BIC. **d** Comparison of the polarization states on Poincaré sphere by enclosing the V point (blue line), C point (green line), and *χ* point (red line). The solid line and dash circles represent the encircling path in upward and downward direction, respectively

Figure 1a shows the typical square-hole silicon PhCS with unperturbed *σ*_*z*_-symmetry and in-plane mirror symmetry standing in free space. The PhCS has a thickness of 600 nm and a lattice constant of 580 nm. The hole has a side length of 330 nm. BIC and topological far-field polarization distribution are expected to be observed in momentum space. This PhCS supports a BIC encompassed by linear polarization symmetrically radiating in upward and downward, as proved by temporal coupled-mode theory (TCMT)^10,33,34^. Breaking in-plane inversion symmetry leads to an undermined BIC and two C points spawning from the original V point at BIC (Fig. 1b). In this case, radiation in upward and downward remains the same, but the polarization states become elliptical. However, the ellipticity angle changes by encircling the C point. By rotating the top hole with an angle of *α*_*top*_, broken *σ*_*z*_-symmetry is introduced in the twisted PhCS (Fig. 1c). The far-field polarization states become elliptical encompassing the central BIC singularity with asymmetric radiation in the upward and downward directions. Figure 1d compares the polarization states on Poincaré sphere for the cases when enclosing the V point, C point and *χ* point. The topological nature of BIC and *χ* point is revealed through the orientation angle of polarization on Poincaré sphere, where the total winding angle of polarization is 2*π* around the central BIC point, indicating a topological charge of +1. It is worth mentioning that this behavior of constant ellipticity around the *χ* point can be used to generate higher-order Poincaré sphere beam, so as to enhance multiplexing capacity of information encryption, optical trapping, and quantum entanglement applications^35–40^.

### Theoretical calculation of *χ*-BIC

Considering finite resonance amplitude and zero incident field in the twisted PhCS, the polarization state of the far-field radiation around BIC is found to be uniquely dependent upon the coupling coefficients from the resonance to the output channels^27,33^

1 $$D = \left( {d_s^u,d_s^d,d_p^u,d_p^d} \right)^T$$

where the superscripts *u* and *d* indicate the upward and downward direction of the PhCS and the subscripts *s* and *p* denote two orthogonal polarization designations.

Each layer of the twisted PhCS can be deemed as a scatter-producing linear polarization at the interfaces, and the slab thickness provides a phase difference ∆Φ between the scatters required for radiating elliptical or circular polarization states^27^. For instance, to radiate far-field circular polarization in upward, the coupling coefficients should fulfill

2 $$\begin{array}{*{20}{c}} {\arg \left( {\frac{{d_s^u}}{{d_p^u}}} \right) = \pm \frac{\pi }{2}} \\ {\frac{{\left| {d_s^u} \right|}}{{\left| {d_p^u} \right|}} = 1} \end{array}$$

From the temporal coupled-mode theory (TCMT), the realization of *χ*-polarized BIC requires (see more details in Supplementary Note 1):

3 $$\begin{array}{*{20}{c}} {\arg \left( {\frac{{d_s^u}}{{d_p^u}}} \right) = \arctan \left[ {\frac{{\tan \left( {2\chi } \right)}}{{\sin \left( {2\psi } \right)}}} \right]} \\ {\frac{{\left| {d_s^u} \right|}}{{\left| {d_p^u} \right|}} = \arctan \left( {\frac{{\sqrt {1 - \cos \left( {2\chi } \right)\cos \left( {2\psi } \right)} }}{{\sqrt {1 + \cos \left( {2\chi } \right)\cos \left( {2\psi } \right)} }}} \right)} \end{array}$$

It is shown that the ellipticity angle *χ* of the BIC can be fully covered by controlling both $d_s^u$ and $d_p^u$ from Eq. 3, which can be realized by tuning the rotation angle of the top structures for the twisted PhCS (see more details in Supplementary Fig. S3).

### Electric field distribution

**Fig. 2:**
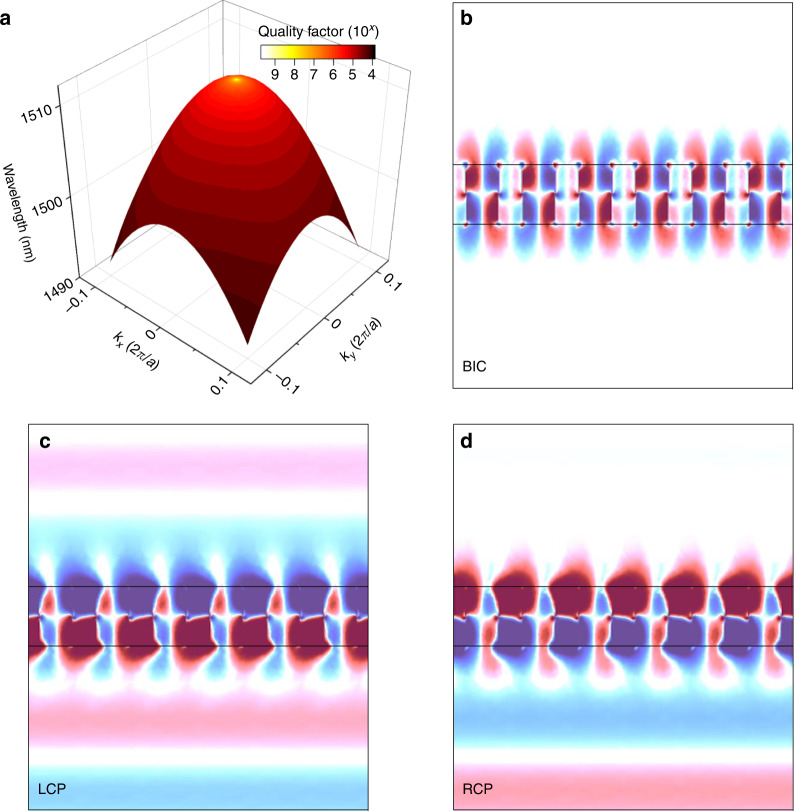
Simulated results of the twisted PhCS supporting BIC with *α*_top_ = 18° and *α*_bot_ = 0°. **a** Iso-frequency surface and color-mapped quality factor of the twisted PhCS. A symmetry-protected BIC is induced at Γ point. **b** Field distribution of the twisted PhCS at Γ point. **c** Left circular polarized field and **d** right circular polarized field at off−Γ point, showing the chiral features of the twisted PhCS

Figure 2a shows the calculated band structure of the twisted PhCS with *α*_*top*_ = 18°, *α*_*bot*_ = 0° and the quality factor of the eigenmode is color-mapped to the iso-frequency surface. The quality factor is not undermined by rotation of the hole, and the twisted PhCS can still maintain the Γ−BIC (detailed in the Supplementary Material) while forming the *χ* point. The bounded resonance at BIC is shown in the field distribution of the twisted PhCS at Γ point (Fig. 2b), where there is no leakage from the PhCS to the upward and downward direction. Chiral feature and asymmetric radiation of the twisted PhCS are demonstrated in left circular polarized field with radiation in both directions (Fig. 2c) and right circular polarized field with only radiation in the downward (Fig. 2d) at off−Γ point.

### Robustness of BIC against rotation of the holes

**Fig. 3:**
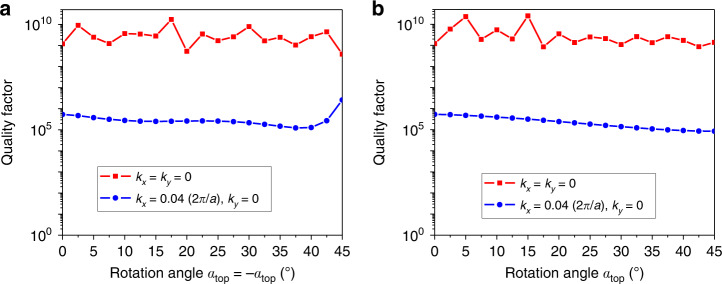
Evolution of the quality factor at BIC and QBIC as the rotation angle increasing from 0° to 45°. **a** is the case with two layers rotates in opposite directions simultaneously. **b** is the case with only the top hole having rotation angle, and the bottom hole is unperturbed

**Fig. 4:**
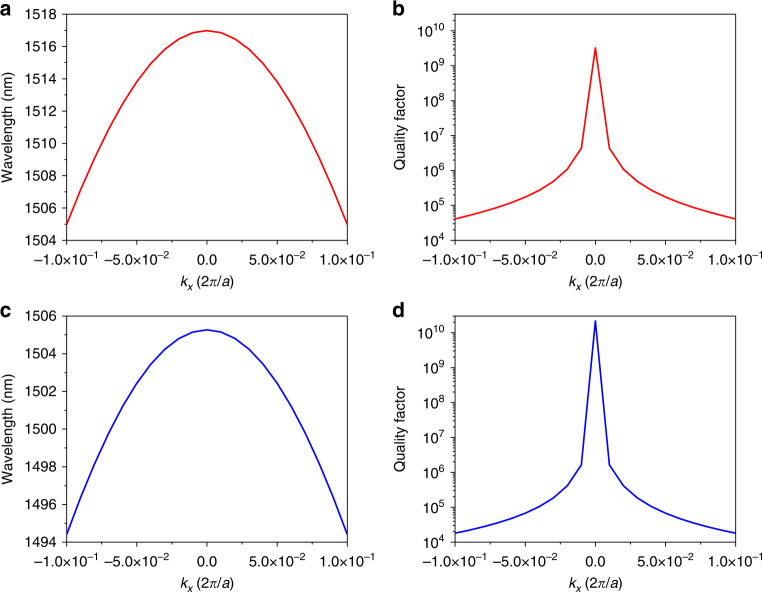
Evolution of BIC eigenmode in the twisted PhCS. Wavelength and quality factor of the eigenmode in the twisted PhCS with (**a**, **b**) *α*_*top*_ = 18° and (**c**, **d**) *α*_*top*_ = 36°. The results are obtained with *k*_*y*_ = 0 and *α*_*bot*_ = 0°

Figure 3 evaluates the robustness of BIC in the PhCS toward rotation of the holes. Figure 3a shows the change of quality factor with response to the rotation angle when the two layers have opposite values *α*_*top*_ = −*α*_*bot*_. The quality factors of both BIC and quasi-BIC remain stable against the perturbation of ration angles. This is the same for PhCS with only top layer having a rotation angle of *α*_*top*_, as shown in Fig. 3b. The quality factor is not undermined by rotation of the hole, and therefore the twisted PhCS can still preserve Γ−BIC. Figure 4 shows the wavelength and quality factor with varying value of wave vector *k*_*x*_ at *α*_*top*_ = 18° (Fig. 4a, b) and *α*_*top*_ = 36° (Fig. 4c, d). BIC in the twisted PhCS is shown with infinite quality factor at *k*_*x*_ = 0.

### Bidirectional *χ*-BIC in upward and downward directions

**Fig. 5:**
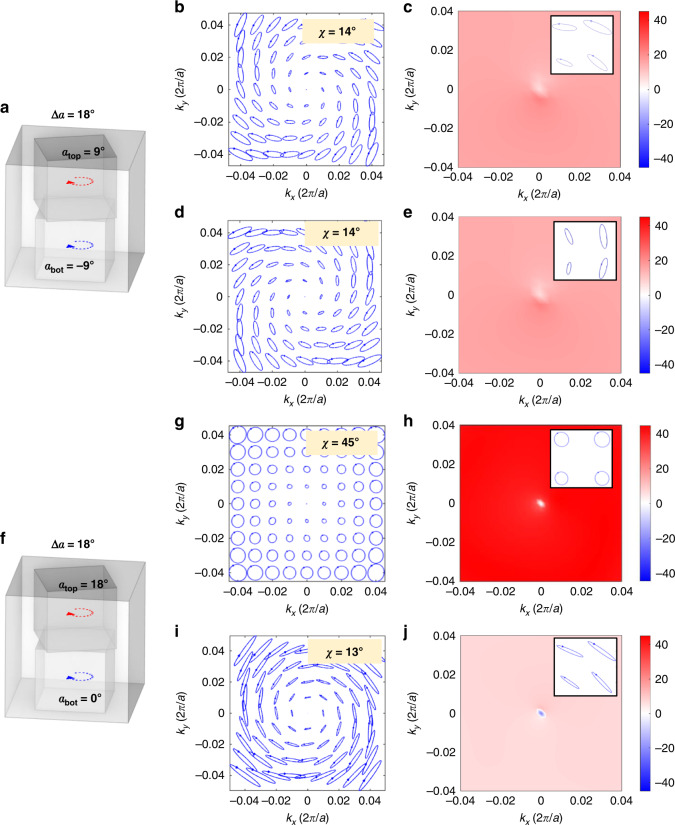
Comparison of far-field polarization states in momentum space of two twisted PhCSs supporting BIC with the same relative angle between top and bottom layer ∆*α* = *α*_top_−*α*_bot_ = 18°. **a** Schematic of the structure with point symmetry against the center of the PhCS at *α*_*top*_ = 9° and *α*_*bot*_ = −9°. Distribution far-field polarization states in (**b**), **c** upward and **d**, **e** downward radiation, showing the same *χ* distribution. **f** Schematic of the PhCS with the same relative angle of ∆*α* = 18° but the different absolute angle of *α*_*top*_ = 18° and *α*_*bot*_ = 0°. **g** distribution of upward far-field polarization states shows circularly polarized radiation at every point in vicinity of BIC. **h** The ellipticity angle (*χ*) of polarization states in (**g**) is 45° indicating circular polarization. **i** Polarization states of downward radiation. **j** The ellipticity angle is 13°, different from upward radiation. Insets in (**h**), **j** show the enlarged view of polarization ellipses

With point symmetry against the center of the PhCS, Fig. 5a illustrates a twisted PhCS having rotation of top hole and bottom hole with relation *α*_*top*_ = −*α*_*bot*_ = 9°. Upward radiation and downward radiating polarization states have the same value of *χ* (Fig. 5b, c for the upward and Fig. 5d, e for the downward radiation), and the polarization orientation angle keeps the charge of 1 (detailed in the Supplementary Material).

To verify the generation of circularly polarized BIC, the rotation angle is fine-tuned to *α*_*top*_ = 18° and *α*_*bot*_ = 0° in Fig. 5f. Figure 5g plots the distribution of upward far-field polarization states, and circular polarization states occur at every point in the vicinity of BIC due to the specially matched phase and amplitude relation for radiation in the *s* and *p* direction based on tunable degree of the broken *σ*_*z*_-symmetry. There is no radiation at BIC as reflected by zero amplitude of polarization ellipse. The corresponding ellipticity angle *χ* of polarization states is extracted in Fig. 5h. Except for a deviation at Γ−BIC point because of its singular feature of undefined polarization state, all other region surrounding the BIC preserves the condition of *χ* = 45°, confirming the realization of a circularly polarized BIC. This phenomenon is in sharp contrast with C points resulting from broken in-plane symmetry as shown in Fig. 1b, where only two circular polarization states occur due to the splitting of BIC vortex point^10^. Here, the BIC point is preserved and there exist constant circularly polarized states just as the linearly polarized states encompassing BIC in conventional PhCS. As a result, projecting the polarization states in the whole Brillouin zone to the Poincaré sphere will give rise to a C point plus a singularity. As for the downward radiation, polarization states become elliptical with different *χ* in Fig. 5i, j. Within the vicinity of BIC, these elliptical polarizations only change their orientation angle *ψ* while preserving the ellipticity angle *χ*. Insets in Fig. h, j illustrate the enlarged view of polarization ellipses appearing in the first quadrant. Projection of the downward polarization states to the Poincaré sphere is then a belt deviated from the L line (or elliptical line) plus a singular point. C points with *χ* = −45° in upward radiation can be approached by conducting mirror symmetry of the twisted PhCS, that is, letting *α*_*top*_ = −18° (detailed in the Supplementary Material). It should be noted that even though the relative rotation angles of the two PhCS layers in both cases of Fig. 5a, f are the same, the absolute values of the rotation angles change the coupling condition between two layers, resulting in different radiating polarization states.

### Full coverage of Poincaré sphere

**Fig. 6:**
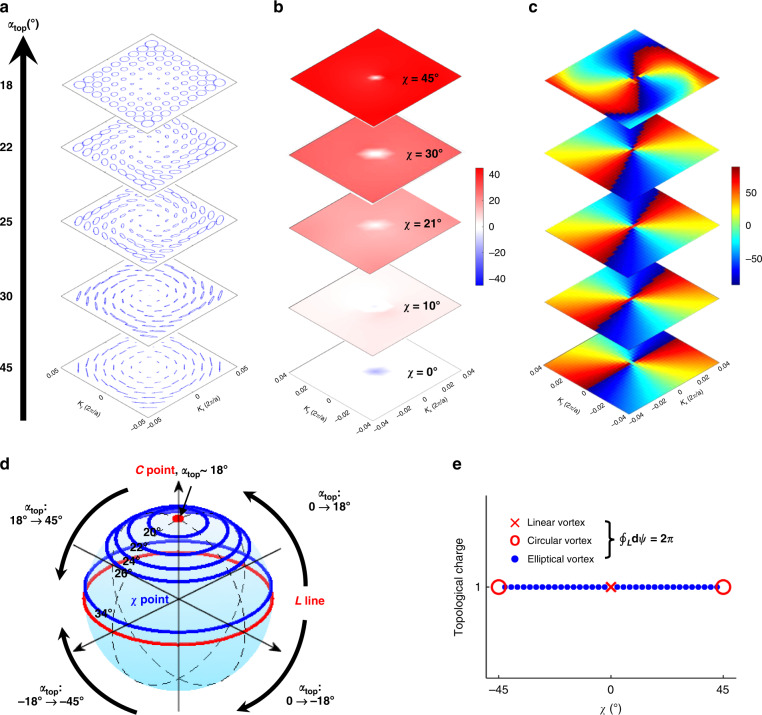
Evolution of far-field polarization states in the upward with varying rotation angle αtop of the top hole in the twisted PhCS. **a** Polarization ellipses evolve from linear state with *α*_*top*_ = 45°, elliptical state with *α*_*top*_ = 45°~18°, to circular state with *α*_*top*_ = 18°. **b** The corresponding values of *χ* show uniform ellipticity in vicinity of each BIC. **c**The orientation angle *ψ* changes from −90° to 90° twice demonstrating BIC topology. **d** Poincaré sphere illustrates the evolution of polarization states around BIC. At every *α*_*top*_, the polarization distribution matches a parallel on the sphere, in accord with different *χ*. Full coverage of Poincaré sphere can be achieved by changing *α*_*top*_. **e** The topological charge maintains 1 for vortex with different *χ*

To better understand the effect of rotation angle *α*_*top*_ on far-field polarization states and demonstrate full coverage of Poincaré sphere assisted by BIC topology, we illustrate the evolution of upward far-field radiation with varying *α*_*top*_ in Fig. 6. Fourfold rotation symmetry of the square PhCS makes it reveal linear polarization states with the rotation angle *α*_*top*_ = 45°, equivalent to the conventional PhCS (Fig. 6a). Decreasing *α*_*top*_ to 30°, 25°, 22° enhances the degree of asymmetry in *z* direction, contributing to elliptical polarization states with increasing value of *χ* as shown in Fig. 6b. Orientation angle *ψ* maintains the topological pattern with winding angle of 2*π* in Fig. 6c regardless of *α*_*top*_. At *α*_*top*_ = 18°, the ellipticity reaches the maximum of 45° and circularly polarized BIC is realized as shown in Fig. 5b. Figure 6d illustrates the topological nature of the arbitrarily polarized BIC by only tuning the rotation angle *α*_*top*_ for coverage of the whole Poincaré sphere (i.e., $- {{\pi \over 4}} \le \chi \le {{\pi \over 4}}$ and $- {\textstyle{\pi \over 2}} \le \psi \le {\textstyle{\pi \over 2}}$). The rotation angle controls uniformly distributed orientation angle *χ* within momentum space near BIC. Since each value of *χ* corresponds to a parallel assisted by BIC topology on the sphere, therefore, by increasing *α*_*top*_ from 0° to 18°, polarization states on the upper hemisphere can be fully covered from linear states (L lines) to circular states (C points). Continuing to increase *α*_*top*_ from 18° to 45°, the far-field polarization gradually changes from C points to L lines. A similar trend holds true for negative *α*_*top*_ that covers the polarization states on the lower hemisphere. The Poincaré sphere can be covered twice with *α*_*top*_ ranging within a span of 90°. This trend can be understood in view of the square’s four-fold rotation symmetry and the difference of polarization angles induced by the two equivalent scatters.

The topological charge carried by BIC with different *χ* can be defined as the winding number of the polarization vortex^13,22^

4 $$q\left( \chi \right) = \frac{1}{{2\pi }}\mathop {\oint}\nolimits_L {d\psi \left( \chi \right)}$$

where *L* is a closed trajectory in momentum space around the BIC of different *χ*. Figure 6e shows the topological charge as a function of *χ*. Arbitrarily polarized BIC can be uniquely defined by the ellipticity angle of polarization states enclosing it. When *χ* = 0°, it is a conventional linearly polarized BIC occurring in the in-plane and *σ*_*z*_- symmetric PhCS. When *χ* has a value between −45° and 45°, it is the elliptically polarized BIC. At *χ* = ±45°, there emerges circularly polarized BIC. Although with circularly polarized BIC the orientation angle *ψ* still reveals topological feature of *q* = 1, the far-filed polarization states are the same since the C point on Poincaré sphere is actually a singular point different from L or elliptical line. This also demonstrates the robustness of BIC topology against an ellipticity angle. Each *χ* point at BIC and its topology correspond to a point on the higher-order Poincaré sphere as proposed in ref. ^35^. By employing the new degrees of freedom in rotation angles of both holes, full coverage of higher-order Poincaré sphere can be achieved with BIC in the twisted PhCS (detailed in the Supplementary Material).

Regarding the fabrication processes, the single-layer PhCS can be fabricated by using conventional e-beam lithography and etching^14,41,42^, followed by direct wafer bonding of two PhCS layers^43^. Furthermore, other techniques using spin-on glass for the fabrication of bilayer silicon structures have also been demonstrated in refs. ^44,45^.

## Conclusion

In conclusion, we reported that by breaking *σ*_*z*_-symmetry of a PhCS, arbitrarily polarized BIC can be realized by controlling the twist angle between two layers of the PhCS. Unlike BIC in unperturbed PhCS or broken in-plane symmetric PhCS where only linear and at most two circular polarization states can occur, the twisted PhCS can exhibit circular polarization states at every point in momentum space within the vicinity of BIC. At different twist angles, other elliptical polarizations can be approached surrounding BIC singular point with the same ellipticity angle. Based on the topological nature of BIC, polarization states on the Poincaré sphere can be fully covered twice with a rotation angle of 90 degrees. Full coverage of the higher-order Poincaré sphere is also attained by tuning the rotation angles of both holes. More diverse polarization control is demonstrated by introducing new degrees of freedom like opposite rotation angle for two layers, breaking both in-plane and *σ*_*z*_- symmetry, and changing the surrounding index. The demonstrated work may contribute to applications of BIC topology in chiral-optical effects, polarization control, structure light like more exotic vector beam, and twistronics.

## Supplementary information

Supplementary Information

## Data Availability

The data that support the findings of this study are available from the corresponding authors upon reasonable request.

## References

[1] Johnson SG (1999). Guided modes in photonic crystal slabs. Phys. Rev. B.

[2] Hsu CW (2016). Bound states in the continuum. Nat. Rev. Mater..

[3] Hsu CW (2013). Observation of trapped light within the radiation continuum. Nature.

[4] Overvig AC (2020). Selection rules for quasibound states in the continuum. Phys. Rev. B.

[5] Zhen B (2014). Topological nature of optical bound states in the continuum. Phys. Rev. Lett..

[6] Jin JC (2019). Topologically enabled ultrahigh-*Q* guided resonances robust to out-of-plane scattering. Nature.

[7] Yang Y (2014). Analytical perspective for bound states in the continuum in photonic crystal slabs. Phys. Rev. Lett..

[8] Doeleman HM (2018). Experimental observation of a polarization vortex at an optical bound state in the continuum. Nat. Photonics.

[9] Zhang YW (2018). Observation of polarization vortices in momentum space. Phys. Rev. Lett..

[10] Liu W (2019). Circularly polarized states spawning from bound states in the continuum. Phys. Rev. Lett..

[11] Bulgakov EN, Maksimov DN (2017). Topological bound states in the continuum in arrays of dielectric spheres. Phys. Rev. Lett..

[12] Yin XF (2020). Observation of topologically enabled unidirectional guided resonances. Nature.

[13] Wang B (2020). Generating optical vortex beams by momentum-space polarization vortices centred at bound states in the continuum. Nat. Photonics.

[14] Huang C (2020). Ultrafast control of vortex microlasers. Science.

[15] Kodigala A (2017). Lasing action from photonic bound states in continuum. Nature.

[16] Song QH (2021). Plasmonic topological metasurface by encircling an exceptional point. Science.

[17] Guo Y, Xiao M, Fan SH (2017). Topologically protected complete polarization conversion. Phys. Rev. Lett..

[18] Liu WZ (2021). Topological polarization singularities in metaphotonics. Nanophotonics.

[19] Che ZY (2021). Polarization singularities of photonic quasicrystals in momentum space. Phys. Rev. Lett..

[20] Chen A (2019). Observing vortex polarization singularities at optical band degeneracies. Phys. Rev. B.

[21] Yoda T, Notomi M (2020). Generation and annihilation of topologically protected bound states in the continuum and circularly polarized states by symmetry breaking. Phys. Rev. Lett..

[22] Ye WM, Gao Y, Liu JL (2020). Singular points of polarizations in the momentum space of photonic crystal slabs. Phys. Rev. Lett..

[23] Zeng YX (2021). Dynamics of topological polarization singularity in momentum space. Phys. Rev. Lett..

[24] Gorkunov MV (2021). Bound states in the continuum underpin near-lossless maximum chirality in dielectric metasurfaces. Adv. Opt. Mater..

[25] Dixon J (2021). Self-isolated raman lasing with a chiral dielectric metasurface. Phys. Rev. Lett..

[26] Gorkunov MV, Antonov AA, Kivshar YS (2020). Metasurfaces with maximum chirality empowered by bound states in the continuum. Phys. Rev. Lett..

[27] Overvig A, Yu NF, Alù A (2021). Chiral quasi-bound states in the continuum. Phys. Rev. Lett..

[28] Hu GW, Qiu CW, Alù A (2021). Twistronics for photons: opinion. Opt. Mater. Express.

[29] Zhao Y, Belkin MA, Alù A (2012). Twisted optical metamaterials for planarized ultrathin broadband circular polarizers. Nat. Commun..

[30] Zhao Y (2017). Chirality detection of enantiomers using twisted optical metamaterials. Nat. Commun..

[31] Chen Y (2022). Multidimensional nanoscopic chiroptics. Nat. Rev. Phys..

[32] Huang L, Zhang WX, Zhang XD (2022). Moiré quasibound states in the continuum. Phys. Rev. Lett..

[33] Hsu, C. W. et al. Polarization state of radiation from a photonic crystal slab. Print at https://arxiv.org/abs/1708.02197v1 (2017).

[34] Fan SH, Suh W, Joannopoulos JD (2003). Temporal coupled-mode theory for the Fano resonance in optical resonators. J. Opt. Soc. Am. A.

[35] Milione G (2011). Higher-order poincaré sphere, stokes parameters, and the angular momentum of light. Phys. Rev. Lett..

[36] Milione G (2015). Using the nonseparability of vector beams to encode information for optical communication. Opt. Lett..

[37] Otte E (2018). Entanglement beating in free space through spin-orbit coupling. Light Sci. Appl..

[38] Shi YZ (2022). Optical manipulation with metamaterial structures. Appl. Phys. Rev..

[39] D’Ambrosio V (2016). Entangled vector vortex beams. Phys. Rev. A.

[40] Qin H (2022). Exploiting extraordinary topological optical forces at bound states in the continuum. Sci. Adv..

[41] Chen ZH (2022). Observation of miniaturized bound states in the continuum with ultra-high quality factors. Sci. Bull..

[42] Song Q (2021). Bandwidth-unlimited polarization-maintaining metasurfaces. Sci. Adv..

[43] Moriceau H (2010). Overview of recent direct wafer bonding advances and applications. Adv. Nat. Sci.: Nanosci. Nanotechnol..

[44] Tanaka K (2020). Chiral bilayer all-dielectric metasurfaces. ACS Nano.

[45] Stolt T (2021). Backward phase-matched second-harmonic generation from stacked metasurfaces. Phys. Rev. Lett..

