# Supplementary material for: Arbitrarily polarized bound states in the continuum with twisted photonic crystal slabs
Source: Light Sci Appl. 2023 Mar 7;12:66. doi: 10.1038/s41377-023-01090-w (PMC9988870; doi:10.1038/s41377-023-01090-w)
Supplement: Supplementary file 1 — Supplementary Information [file 41377_2023_1090_MOESM1_ESM.pdf]

Supplementary Information

Arbitrarily Polarized Bound States in the Continuum with Twisted Photonic Crystal Slabs

Haoye Qin[1], Zengping Su[1], Mengqi Liu[2], Yixuan Zeng[2], Man-Chung Tang[1], Mengyao Li[1], Yuzhi Shi[3], Wei Huang[4], Cheng-Wei Qiu[2*], Qinghua Song[1†]

[1]Tsinghua Shenzhen International Graduate School, Tsinghua University, Shenzhen, 518055, China

[2]Department of Electrical and Computer Engineering, National University of Singapore, 117583 Singapore

[3]Institute of Precision Optical Engineering, School of Physics Science and Engineering, Tongji University, Shanghai 200092, China

[4]Key Laboratory of Multifunctional Nanomaterials and Smart Systems, Suzhou institute of Nano-Tech and Nano-Bionics (SINANO), Chinese Academy of Sciences (CAS), Suzhou, 215123 China

*Corresponding Authors: *chengwei.qiu@nus.edu.sg*

†song.qinghua@sz.tsinghua.edu.cn

Supplementary Note 1: Analysis of polarization states radiating from the twisted PhCS with temporal coupled-mode theory

The dynamics of a resonance in the twisted PhCS on an isolated band can be described phenomenologically with temporal coupled-mode theory (TCMT)[1-3]

$$\frac{dA}{dt} = (-i(\omega - \omega_0) - \gamma)A + D^T s^+ \quad (1)$$

$$s^- = Cs^+ + DA \quad (2)$$

where A is the resonance amplitude, ω is the angular frequency, ω_0 is the resonant angular frequency, γ is the damping rate due to radiative loss. s^+ and s^- represents incident and outgoing wave amplitudes, respectively. C is the background scattering matrix describing the direct (non-resonant) scattering response. Column vector D contains the coupling coefficients from the resonance to the input/output channels

$$D = \left(d_s^u, d_s^d, d_p^u, d_p^d\right)^T \quad (3)$$

where the superscripts u and d indicate the upward and downward direction of the PhCS and the subscripts s and p denote two orthogonal polarization designations. The overall scattering matrix can be written as,

$$S = C + \frac{DD^*}{i(\omega - \omega_0) + \gamma} \quad (4)$$

As proven in Ref. [3], by considering a finite amplitude in the resonance and no incident field $s^+ = 0$, the outgoing radiation from the eigenmode is $s^- = DA$. This indicates that the polarization state of the far-field radiation is uniquely dependent upon the coupling coefficients D. The polarization states radiating in the upward are related to the amplitude ratio and phase difference between d_s^u and d_p^u. For the downward radiation, they are related

to the amplitude ratio and phase difference between d_s^d and d_p^d. Circular polarized states enclosing BIC can be find by minimizing the value degree of circular polarization

$$DOC = 10log_{10}\left[\frac{4}{\pi^2}\left(\arg\left(\frac{d_s^u}{d_p^u}\right) \pm \frac{\pi}{2}\right)^2 + \left(\frac{|d_s^u|}{|d_p^u|} - 1\right)^2\right] \tag{5}$$

Each layer of the twisted PhCS can be deemed as a scatter producing linear polarization coupled to free space, the angle of which depends on the rotation angle of the hole. We assume the top (bottom) layer has the polarization angle of $\phi(\theta)$. The top surrounding-PhCS interface is denoted as port 1 and the bottom as port 2. There should be a difference in coupling coefficients between bottom scattering to port 1 (or top scattering to port 2, indirectly coupling, defined as c_{ind}) and top scattering to port 1 (bottom scattering to port 2, directly coupling, defined as c_{dir}) due to the thickness of PhCS in *z* direction[4]. Then the coupling coefficients *D* is given in the form of

$$\begin{aligned} d_s^u &= c_{dir}\cos(\phi) + c_{ind}\cos(\theta) \\ d_s^d &= c_{ind}\cos(\phi) + c_{dir}\cos(\theta) \\ d_p^u &= c_{dir}\sin(\phi) + c_{ind}\sin(\theta) \\ d_p^d &= c_{ind}\sin(\phi) + c_{dir}\sin(\theta) \end{aligned} \tag{6}$$

The directly coupling and indirectly coupling are related with $c_{ind} = ge^{i\Delta\Phi}c_{dir}$, where g is the amplitude ratio and $e^{i\Delta\Phi}$ reflects the phase difference between the two scattering processes. In order to realize circular polarization distribution for upward radiation shown in Fig. S1(b) and Fig. 2 in the main text, the requirements of $\arg\left(\frac{d_s^u}{d_p^u}\right) = \pm\frac{\pi}{2}$ and $\frac{|d_s^u|}{|d_p^u|} = 1$ should be fulfilled. For an unperturbed bottom hole, the scattering polarization angle θ is assumed to be zero, leading to a simplified result

$$\begin{aligned} \cos(\phi) &= -g\cos(\Delta\Phi) \\ |\sin(\phi)| &= \left|\cos(\phi) + ge^{i\Delta\Phi}\right| \end{aligned} \tag{7}$$

with $g = -\frac{\sqrt{1-r^2\cos^2(\Delta\Phi)^2} - |r|\sin(\Delta\Phi)}{\sqrt{1-r^2}}$ and r is the reflection rate in the background scattering matrix. While to realize χ-polarized BIC, the phase difference (δ) and amplitude ratio between two items represented in Jones vector are remained to be extracted. In view of the relation of Stokes parameters, the following relation applies[5],

$$\begin{gathered} S_0 + S_1 = 2E_{ox}^2 \\ S_0 - S_1 = 2E_{oy}^2 \\ S_2 = 2E_{ox}E_{oy}\cos(\delta) = S_0\sin(2\psi)\cos(2\chi) \\ S_3 = 2E_{ox}E_{oy}\sin(\delta) = S_0\sin(2\psi) \end{gathered} \tag{8}$$

Therefore, the phase difference and amplitude ratio for χ-polarized BIC are given as

$$\begin{gathered} \arg\left(\frac{d_s^u}{d_p^u}\right) = \arctan\left[\frac{\tan(2\chi)}{\sin(2\psi)}\right] \\ \frac{|d_s^u|}{|d_p^u|} = \arctan\left(\frac{\sqrt{1-\cos(2\chi)\cos(2\psi)}}{\sqrt{1+\cos(2\chi)\cos(2\psi)}}\right) \end{gathered} \tag{9}$$

The value $\Delta\Phi$ is a phenomenological parameter that depends on the specific structure and surrounding of PhCS[4]. We can estimate the value of $\Delta\Phi$ for twisted PhCS with unperturbed bottom hole by comparing the position of upward circularly polarized BIC obtained from TCMT and simulations. Fig. S2(a) shows the result of DOC as a function of $\Delta\Phi$ and ϕ The circularly polarized states are represented by the blue regions. Considering the four-fold rotation symmetry of square, the relation between geometric parameter α_{top} and ϕ in TCMT can be obtained as $\phi = 4\alpha_{top}$. This is also verifed in Fig. S2 (a), where the DOC goes to over 30 dB as linear polarization at $\phi = -180, 0, 180°$, corresponding to linearly polarized BIC obtained at $\alpha_{top} = -45, 0, 45°$ with simulation. Fig. S2 (b) shows the calculated DOC from simulation as a function of thickness. The linear regions agree well with TCMT, and the circular regions are undermined when changing thickness away from 600 nm. This can be attributed to that not only the thickness but also rotation angle α_{top}

will change the value $\Delta\Phi$. Since circularly polarized BIC is obtained at $\alpha_{top} = 18°$ in simulation, the value of $\Delta\Phi$ should equal to 202º for the DOC going to the minimum at $\phi = 4\alpha_{top} = 72°$.

The results of TCMT analysis and COMSOL simulation for the twisted PhCS with both layers having a rotation angle with the obtained $\Delta\Phi$ agree well with each other as shown in Fig. S3. At $\alpha_{bot} = 0°$, the simulation agrees well with the TCMT, with the minimum points (circular polarizations) both occur at $\alpha_{top} = \pm 18°$ and the peaks at $\alpha_{top} = \pm 45, 0°$ (Fig. S3 (a-b)). When increasing the rotation angle of the bottom hole to $50°$, the lineshapes obtained from simulation and TCMT have similar trend for the positions of peaks and dips (Fig. S3 (c-d)). However, some deviations like the minimum values and peak values occur due to the fact that changing α_{bot} also affects the value of $\Delta\Phi$ to some extent. The simulated result of sweeping α_{top} and α_{bot} is demonstrated in Fig. S4. Seven symmetric red regions are linear polarizations at $(\alpha_{top}, \alpha_{bot}) = (\pm 45°, 0°)$, $(\alpha_{top}, \alpha_{bot}) = (0°, \pm 45°)$, $(\alpha_{top}, \alpha_{bot}) = (0°, 0°)$, and $(\alpha_{top}, \alpha_{bot}) = (\mp 20°, \pm 20°)$. While circular polarizations are found to appear only at $\alpha_{bot} = 0°$, indicating the perturbation of α_{bot} to $\Delta\Phi$.

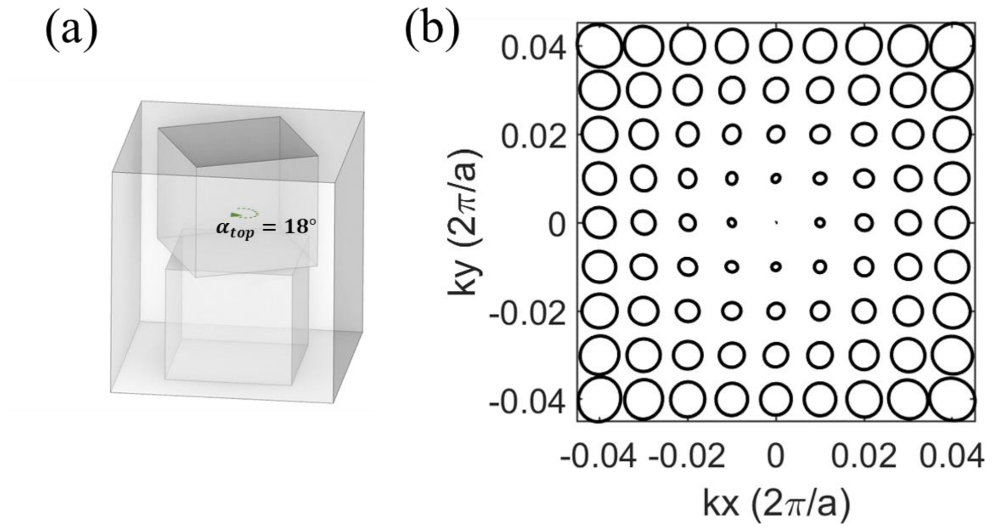

Figure S1. Structure design of the circularly polarized BIC in twisted PhCS. (a) Twisted PhCS structure with the top hole rotated with an angle of $\alpha_{top} = 18°$. (b) Far-field polarization radiating in the upward direction in momentum space.

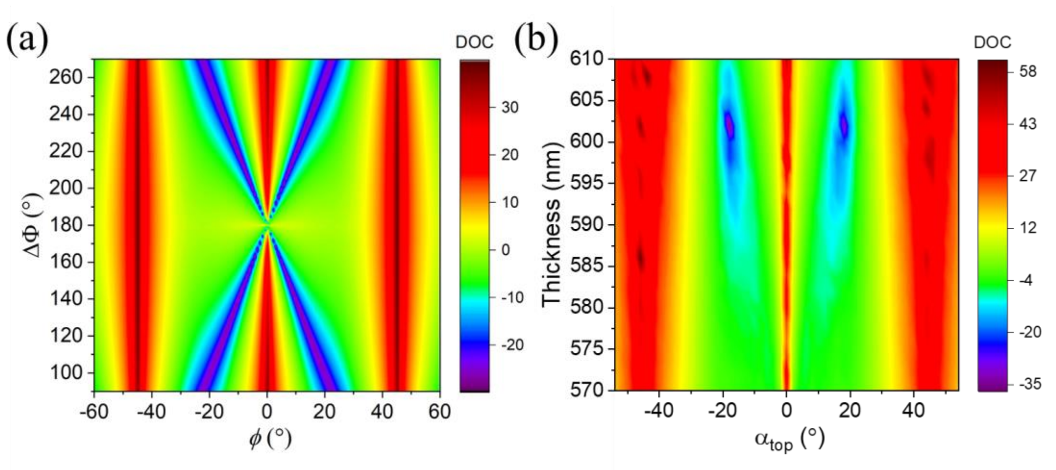

Figure S2. (a) DOC as a function of $\Delta\Phi$ and ϕ calculated from TCMT. (b) DOC as a function of geometric parameters thickness and α_{top} obtained from simulation.

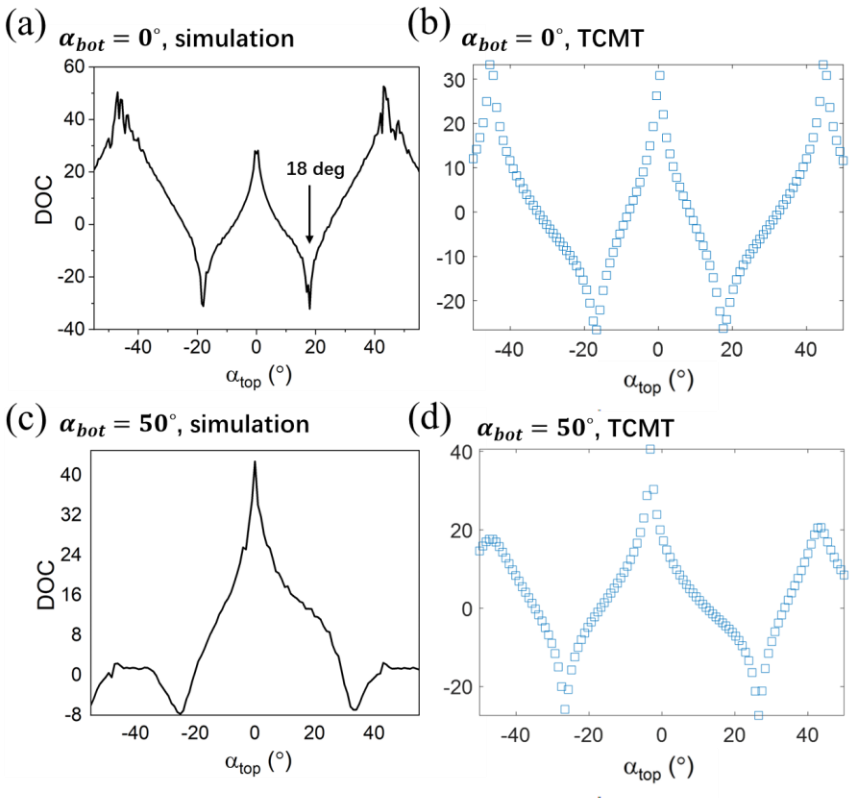

Figure S3. Comparison between DOC results from TCMT and simulation for varying rotation angle of the bottom hole α_{bot}. (a) Simulated and (b) TCMT predicted results with $\alpha_{bot} = 0°$. (c) Simulated and (d) TCMT predicted results with $\alpha_{bot} = 50°$.

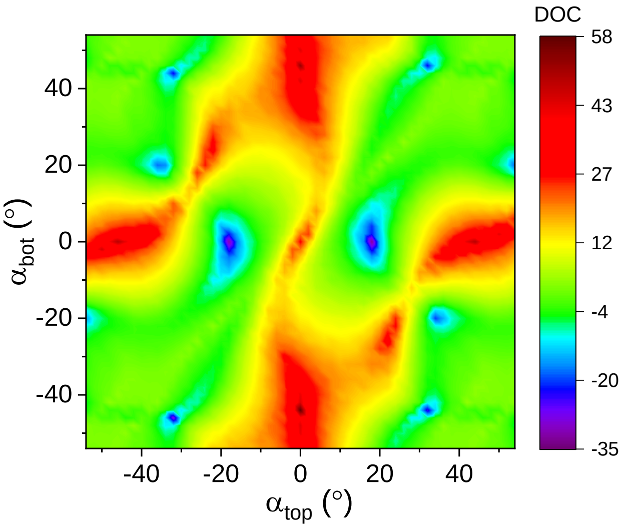

Figure S4. Simulated DOC as a function of rotation angle of the two holes.

Supplementary Note 2: Circular polarized BIC with negative rotation angle

For upward far-field radiation, the polarization states will have negative ellipticity angle χ when $\alpha_{bot} = 0°$. Fig. S5 demonstrates simulated results with the rotation angle of $\alpha_{top} = -18°, -10°, -20°$. As expected, in Fig. S5 (a-b) with $\alpha_{top} = -18°$, the far-field polarization states become circular polarizations at every point within vicinity of BIC with $\chi = -45°$. Fig. S5 (c-f) have elliptical polarizations equipping with $\chi = -18°$ and $\chi = -38°$. For circularly polarized BIC, negative and positive rotation will have opposite χ, which can be understood from both geometric symmetry and TCMT. By letting θ = 0, the requirement of $\arg\left(\frac{d_s^u}{d_p^u}\right) = \pm\frac{\pi}{2}$ can be reduced to

$$\frac{\cos(\phi)+ge^{i\Delta\Phi}}{\sin(\phi)} = \pm i \tag{10}$$

where the denominator of left term is an odd function, implying $\alpha_{bot} = \pm 18°$ will have opposite χ.

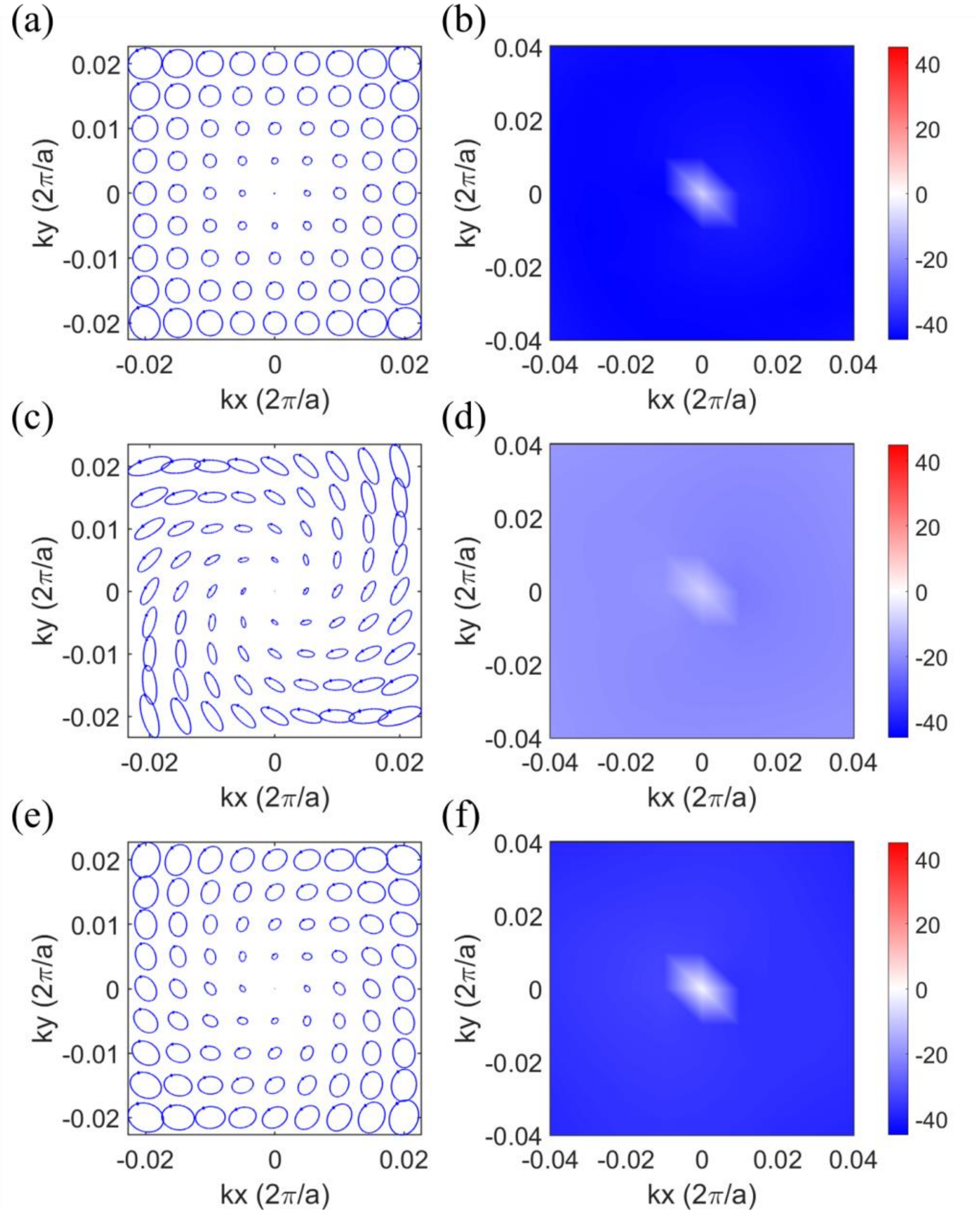

Figure S5. (a,c,e) Far-field polarization states with $\alpha_{top} = -18°, -10°, -20°$ in momentum space. (b,d,f) Corresponding ellipticity angle of polarization.

Supplementary Note 3: Breaking both in-plane inversion symmetry and σ_z symmetry

As demonstrated in Ref. [6], by breaking the in-plane inversion symmetry of a square PhCS, pairs of circularly polarized states will spawn from the V point of the eliminated BIC. Fig. S6 show the results with making the square become trapezoid of $L1/L2$ = 0.8. Fig. S6 (a) is the schematic of the structure with no twist. Fig. S6 (b) is the far-field polarization states with two C points spawning from the BIC. The circular polarization states are verified in Fig. S6 (c) with extracted ellipticity angle of $\pm 45°$. Both C points are half-charged as shown in map of orientation angle in Fig. S6 (d).

By introducing both in-plane inversion symmetry and σ_z symmetry, the V point will break up into two C points with charge of 1/2 that also rotate in the momentum space as shown in Fig. S7. The broken in-plane symmetry results in splitting BIC vortex point with charge of 1 to two C points distributed symmetrically at $k_y = 0$, and the broken σ_z symmetry leads to rotation of the two C points with $\chi = \pm 45°$, as illustrated by the tilted dashed line (Fig. S7(a)). Increasing the rotation angle can continuously manipulate positions of the two C points and the related topological polarization distribution in a circle (indicated by the arc arrows, detailed in the Supplementary Material). Fig. S7 (b,d,f) show the far-field polarization states when increasing the rotation angle oppositely from $|\alpha_{top}| = |\alpha_{bot}| = 16°$ to $28°$, and Fig. S7 (c,e,g) are the corresponding ellipticity angles. C points are represented by $\chi = \pm 45°$. The two C points are observed to rotate in momentum space with the increasing rotation angle of both holes.

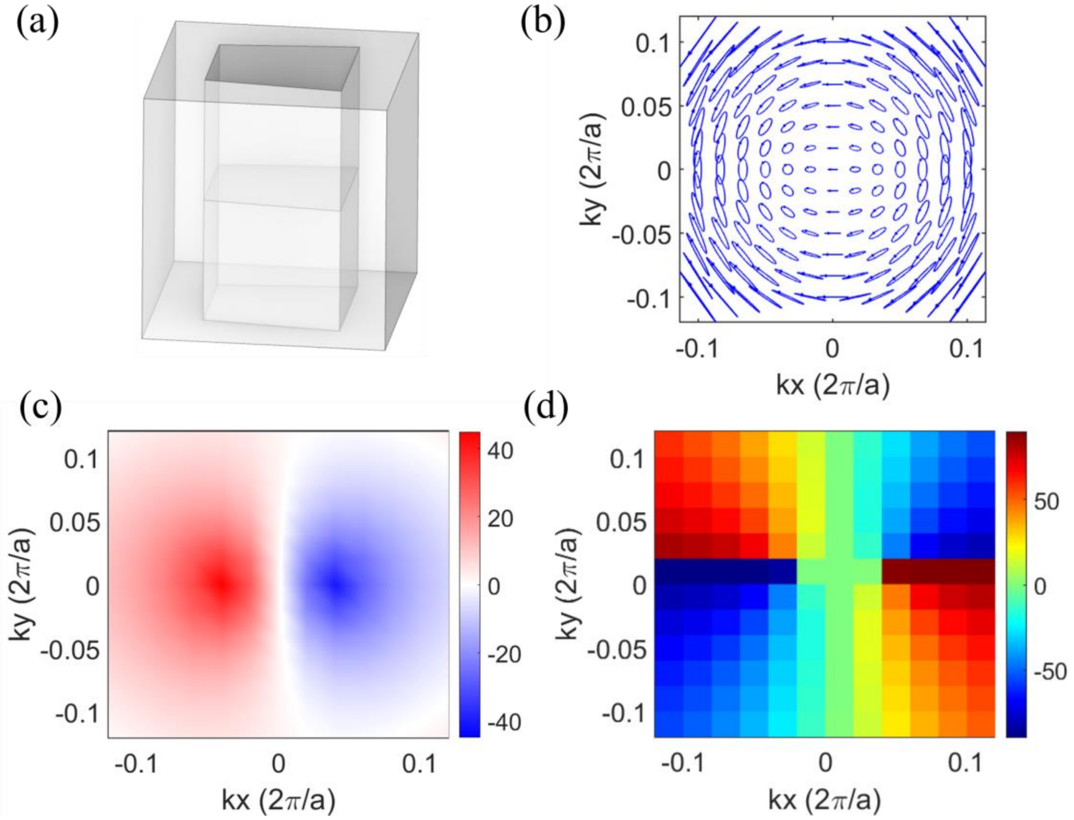

Figure S6. Broken in-plane inversion symmetry of a PhCS. (a) Schematic of the PhCS with trapezoid hole of L_1/L_2 = 0.8. (b) Far-field polarization states with two C points spawning from BIC. (c) Map of ellipticity angle. (d) Map of orientation angle.

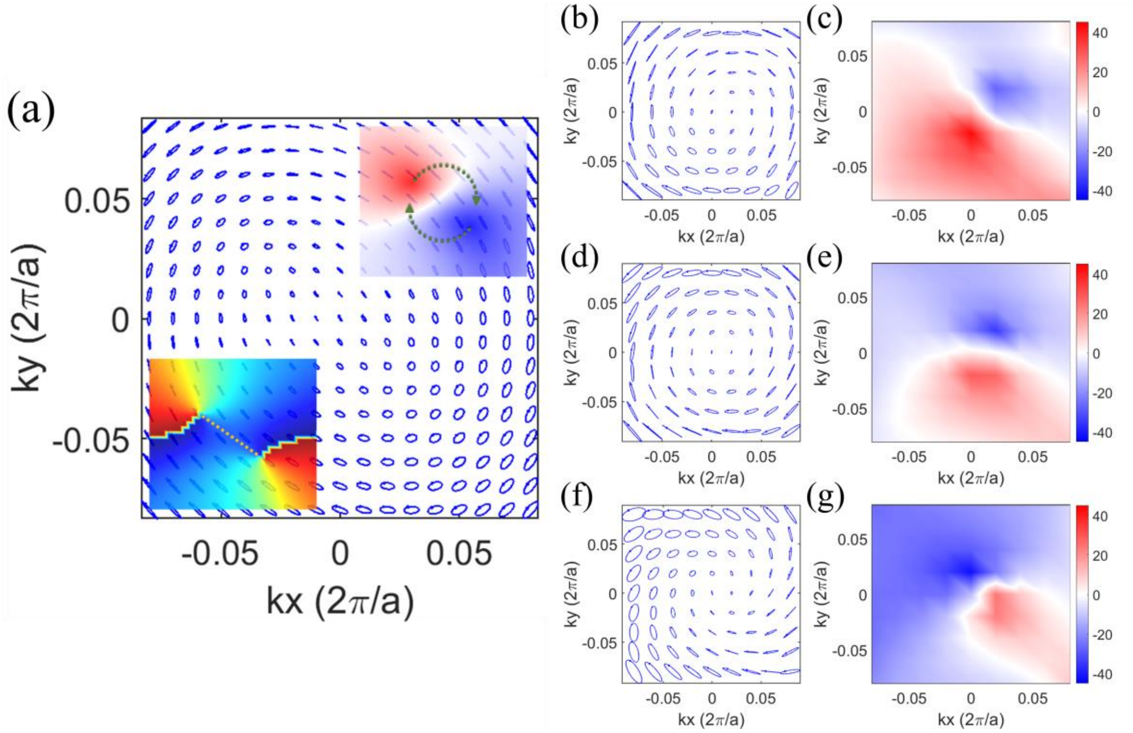

Figure S7. Far-field polarization states in the momentum space with broken both in-plane inversion symmetry and σ_Z symmetry. (a) Polarization states and maps of χ, ψ. The length ratio L_1/L_2 is 0.8 and $\alpha_{top} = -\alpha_{bot} = 16°$ (b,d,f) Polarization states with $\alpha_{top} = 16°, 22°, 28°$. (c,e,g) Corresponding ellipticity angle of polarization shows twisted effected around the two half-charge C points.

Supplementary Note 4: Polarization features of a continuously twisted PhCS

Unlike the discrete twist demonstrated previously, the PhCS can also be twisted continuously as illustrated in Fig. S8 (a), which also breaks the σ_z symmetry. The corresponding far-field polarization states are demonstrated in Fig. S8 (b-d), having a cyclone-like distribution in momentum space. BIC and its topology are maintained. There are four regions with negative χ in contrast with the central positive-χ region.

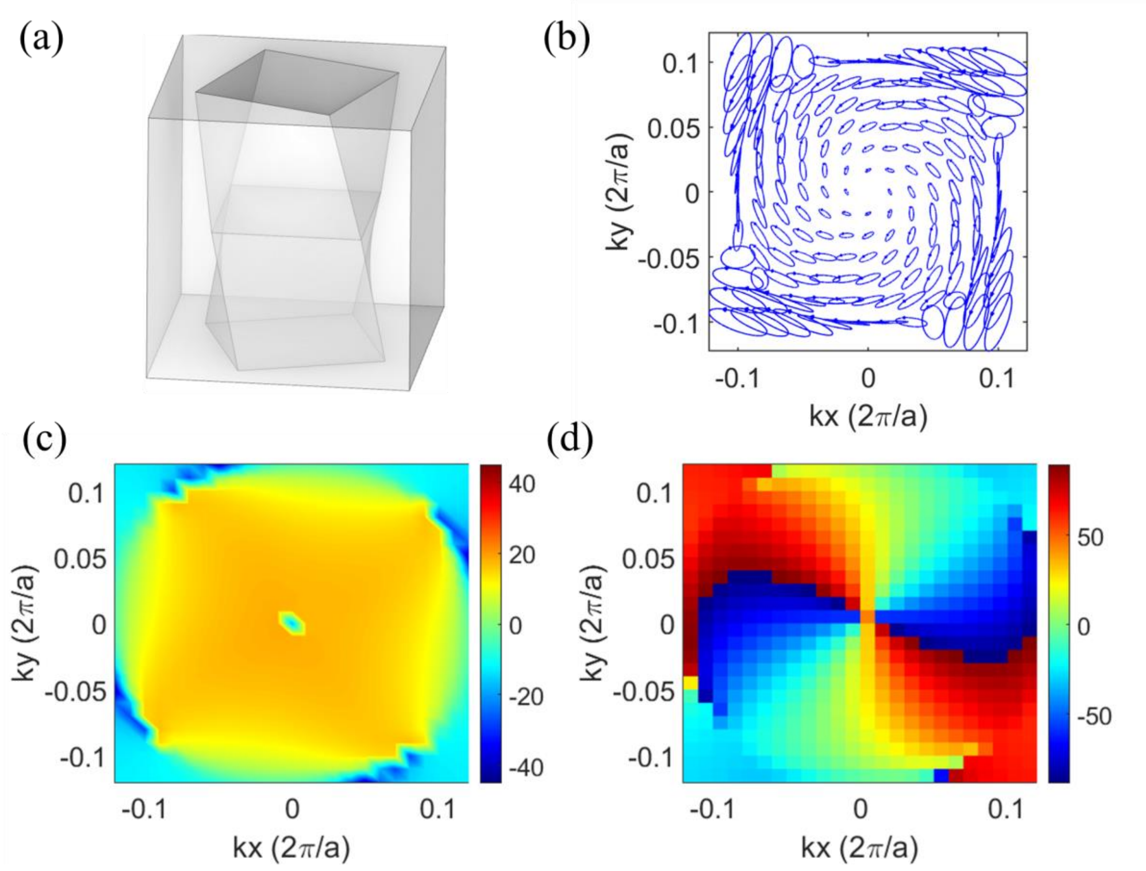

Figure S8. Polarization states from a continuously twisted PhCS. (a) Schematic of the PhCS with continuously twist angle of 20°. (b) Far-field polarization states in momentum space. (c) Map of ellipticity angle. (d) Map of orientation angle.

Supplementary Note 5: Radiation of circular polarizations in both directions

By tuning the thickness of PhCS and rotation angle of the holes, far-fled polarization can both equip with circular polarizations. Fig. S9 show the far-field polarizations within a large range in momentum space. Fig. S9 (a,b) show the upward radiation of circular polarizations and Fig. S9 (c,d) is the downward, with PhCS thickness tuned to 530 nm and $\alpha_{top} = -\alpha_{bot} = 25°$. BIC is lost on this band. The radiation power is nearly on the same scale with no singular point. Circular polarizations are observed to be distorted outside the dashed circle due to a very large distance away from Γ point.

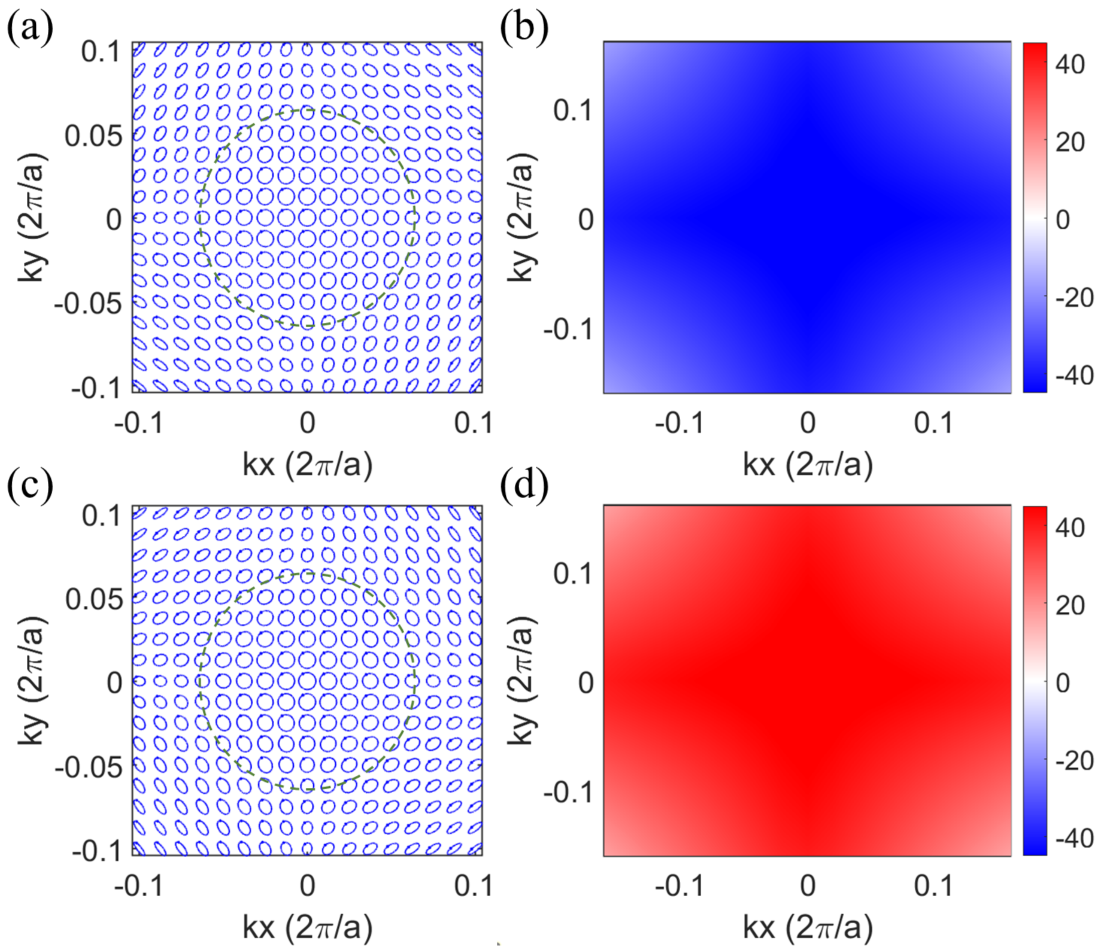

Figure S9. (a,c) Polarization states and (b,c) ellipticity angles of the eigenmode in the twisted PhCS with $\alpha_{top} = -\alpha_{bot} = 25°$ and PhCS thickness of 530 nm.

Supplementary Note 6: Topology of BICs on different bands

Figure S10 demonstrate the topology of downward polarization states around BICs at two bands in the unperturbed PhCS. In Fig. S10 (a), the frequency iso-surfaces show that BIC with infinite quality factor is found separately with two eigenmodes. Fig. S10 (b-d) plot the polarization ellipticity angle (χ), orientation angle (ψ), and angle of the polarization vector for the upper surface[7]. Fig. S10 (e-g) show the relevant results for the lower surface. The two bands contain opposite topological charge as revealed from the orientation angle and angle of the polarization vector. The polarization vectors on the two bands will have an orientation angle of 90° difference[8].

Figure S11 demonstrates the far-field polarization states for the lower band in a PhCS with $\alpha_{top} = 9°$ and thickness of 555 nm. The ellipticity angle of polarization states remain nearly stale in Fig. S11 (a,b). Topological features of BIC are revealed in Fig. S11 (c,d) showing the orientation angle and angle of the polarization vector.

Figure S12 demonstrate the robustness of quality factor at BIC on the two bands against broken σ_z symmetry. BIC on the lower band in both Fig. S12 (a) with rotation of two layers and Fig. S12 (b) with rotation of the top hole is distorted at some degrees. This is attributed to that the field profile of the lower band's eigenmode locates around the hole, while the upper band has mode distribution around the edges of silicon.

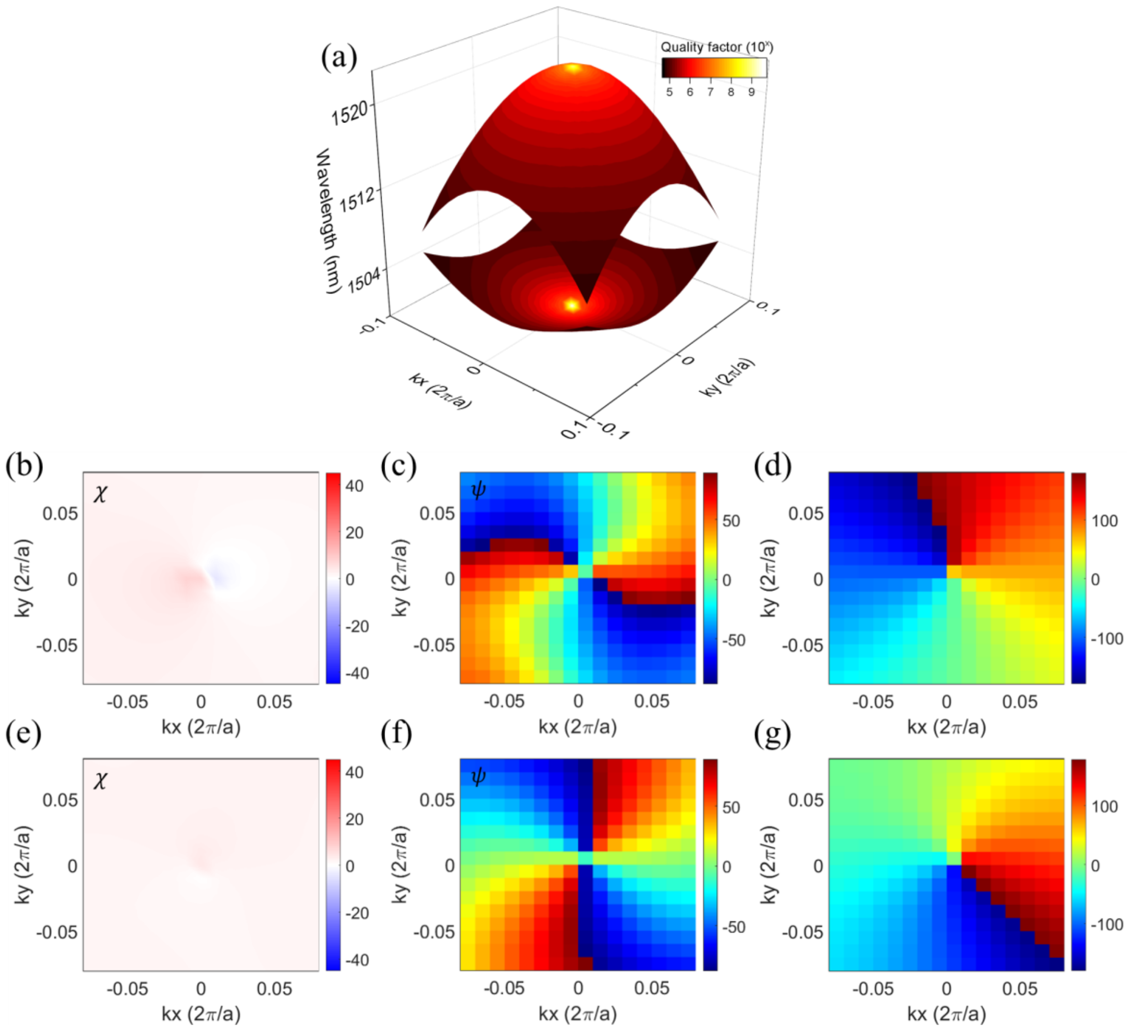

Figure S10. (a) Iso-frequency surface and color-mapped quality factor of two bands showing BIC in the unperturbed PhCS. (b,e) Map of polarization ellipticity angle. (c,f) Map of polarization orientation angle. (d,g) Map of angle of the polarization vector. (b-d) and (e-g) correspond to the upper band and lower band, respectively.

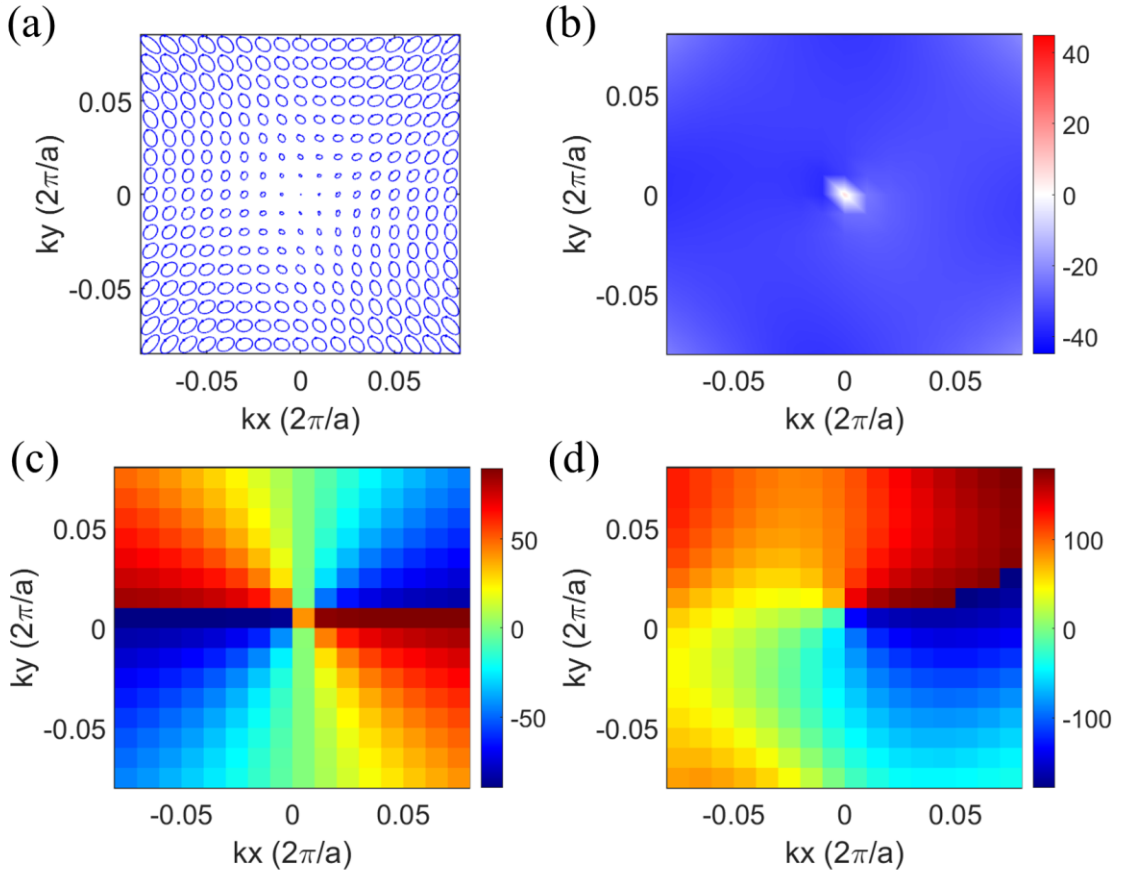

Figure S11. Far-field polarization states around BIC on the lower band in Fig. S12. (a) in a PhCS with $\alpha_{top} = 9°$ and thickness of 555 nm. (a) Polarization states around the BIC. (b) Map of polarization ellipticity angle. (c) Map of polarization orientation angle. (d) Map of angle of the polarization vector.

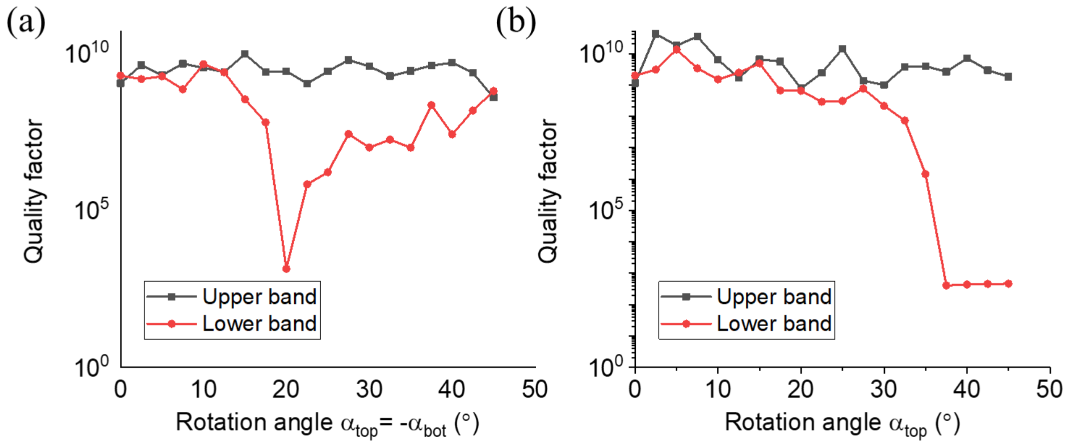

Figure 12. Evolution of the quality factor at BIC on the two bands as the rotation angle increasing from 0° to 45°. (a) Two layers rotates in opposite directions simultaneously. (b) Only the top hole has rotation angle, and the bottom hole is unperturbed.

Supplementary Note 7: Robustness of the twisted PhCS for circularly polarized BIC against surrounding index.

To demonstrate the robustness and fabrication consideration of the twisted PhCS for supporting circularly polarized BIC, Fig. S13(a-c) evaluate the upward polarization state in the vicinity of BIC as a function of PhCS thickness and rotation angle with the surrounding material index n_{sur} = 1.1, 1.3, 1.5. In case of immersing the twisted PhCS in other materials like index-matching liquid or glass[9,10], circular BIC can still be approached by a compensation between PhCS thickness and rotation angle shown with dark-blue region. The required value of αtop is found to decrease with increasing n_{sur}.

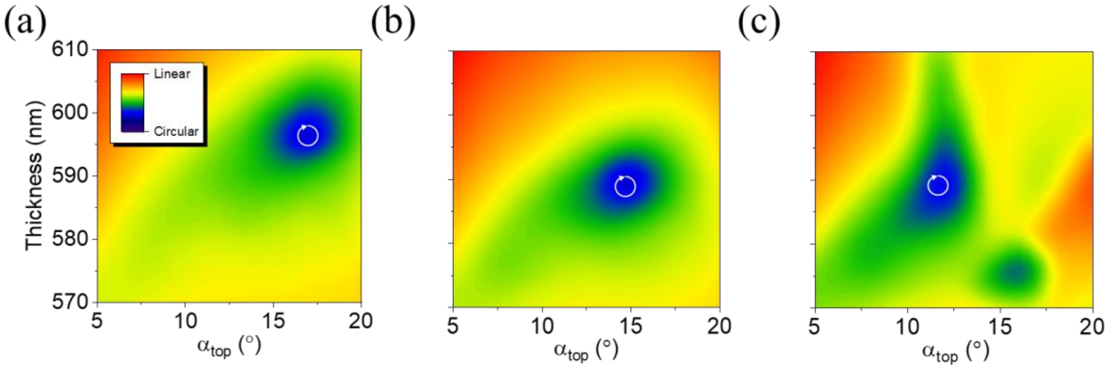

Figure S13. At $\alpha_{top} = 0°$, parameter selection of α_{top} and slab thickness for generating circularly polarized BIC for upward radiation when the surrounding index (n_{sur}) changes to (a) 1.1, (b) 1.3, (c) 1.5. The dark blue color indicates circular polarization.

Supplementary Note 8: Asymmetric radiation of polarization states in the upward and downward direction.

A twisted PhCS will generally show asymmetry radiation in the upward and downward direction due to its broken-σ_z symmetry and difference of scattering angle between two layers. Fig. S14 (a,b) demonstrate the asymmetric radiation of polarization states characterized by ΔDOC defined as the difference between DOC of the upward and downward radiation.

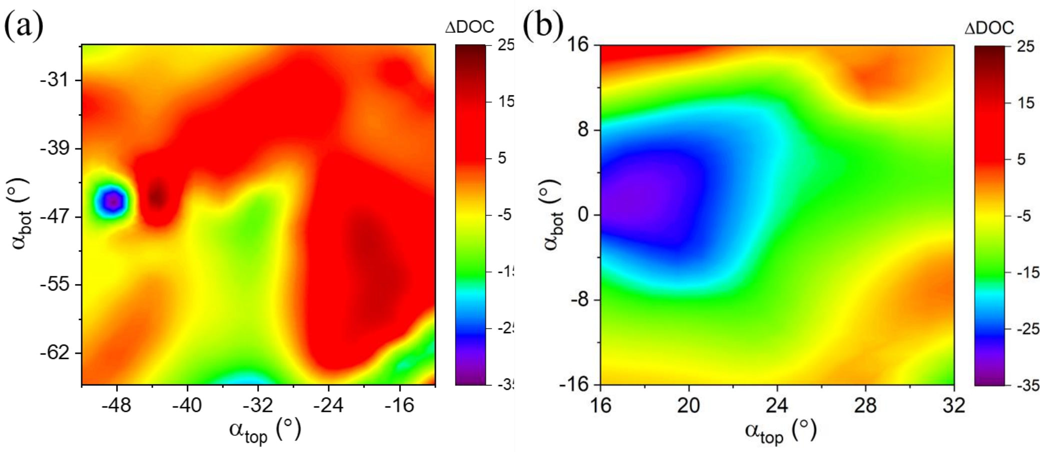

Figure S14. (a,b) Asymmetric radiation of polarization states characterized by ΔDOC.

Supplementary Note 9: Rotation of holes without vertical displacement.

For the PhCS structure with rotation of holes without vertical displacement illustrated in Fig. S15 (a), quality factor at BIC is preserved with infinite lifetime (Fig. S9 (b)). At a rotated angle of 30°, the orientation angle in Fig. S15 (c) has a topological charge of 1, confirming the BIC topology. Since there is no broken-σ_z symmetry, the polarization states remain linear in Fig. S15 (d-f) with different rotation angles, in contrast with polarization states in the main text. The rotation changes distribution of the orientation angles while keeping the topological charge.

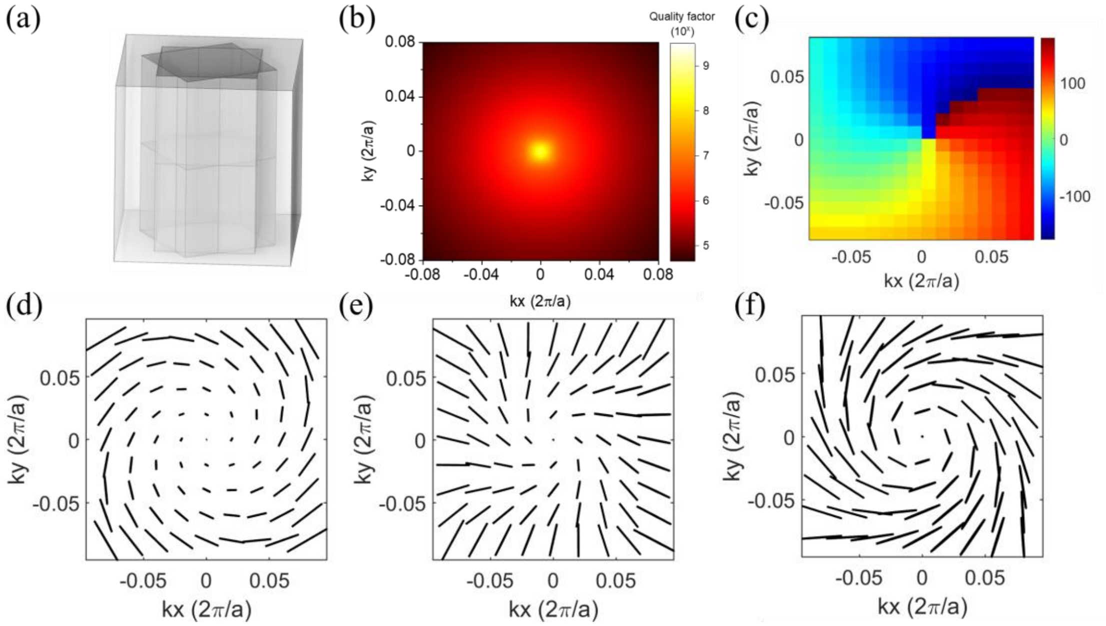

Figure S15. Polarization states around BIC in a PhCS with rotated holes without vertical displacement. (a) Schematic of the PhCS with rotated holes without vertical displacement. (b) Quality factor of the BIC in the PhCS with rotated holes without vertical displacement. (c) Orientation angle of polarization states with one hole having a rotation angle of 30°. (d-f) Far-field polarization states with one hole having a rotation angle of 20°, 30°, and 40°.

Supplementary Note 10: Full coverage of higher-order Poincaré sphere.

As illustrated in Ref. [11], each χ point at BIC and its topology induced polarization winding (the orientation angle ψ) correspond to a point on the higher-order Poincaré sphere. Fig. S16 shows the representation of χ point at BIC on the higher-order Poincaré sphere with varying α_{bot} from $-45°$ to $45°$ at a specific $\Delta\alpha$. Fig. S16(a) shows the evolution of the χ point at $\Delta\alpha = 18°$ and the trend crosses a pole on the higher-order Poincaré sphere, indicating a topological distribution of circular polarization states. When there is no rotation difference $\Delta\alpha = 0°$, the equator can be covered by changing the rotation angle in Fig. S16(b). Similar pattern holds true for other values of $\Delta\alpha$ like Fig. S16(c) with $\Delta\alpha = 22°$. By sweeping the rotation angle of both holes (α_{bot} and $\Delta\alpha$), full coverage of higher-order Poincaré sphere can be achieved with BIC in the twisted PhCS (Fig. S16(d)). The full lower hemisphere can be covered with varying $\Delta\alpha$ from 0° to $45°$. Considering the symmetry, full coverage of the upper hemisphere can be covered with varying $\Delta\alpha$ from0° to $-45°$.

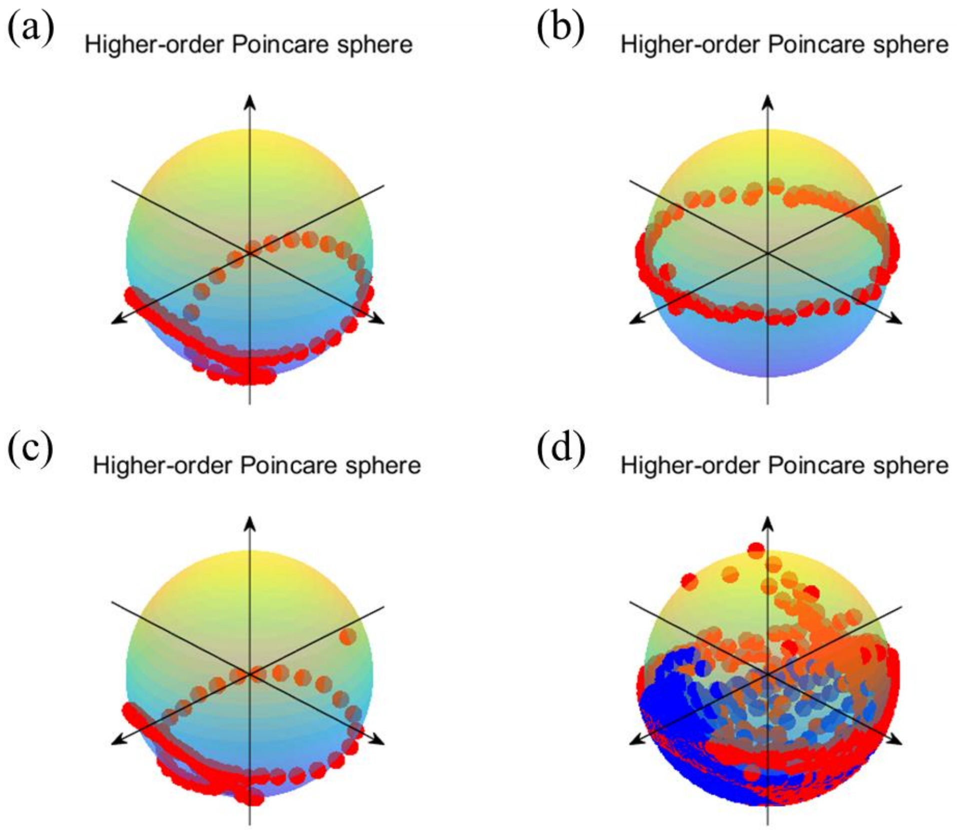

Figure S16. Representation of χ point at BIC on the higher-order Poincaré sphere. The rotation angle of the bottom hole $\boldsymbol{\alpha_{bot}}$ is varying from $\mathbf{-45°}$ to $\mathbf{45°}$ with a specific $\boldsymbol{\Delta\alpha}$. (a) $\Delta\alpha = 18°$. (b) $\Delta\alpha = 0°$. (c) $\Delta\alpha = 22°$. (d) The value of $\Delta\alpha$ is $0° - 18°$ for the red dots and $18° - 45°$ for the blue dots.

Supplementary Note 11: Impact of gap between two layers.

In consideration of fabrication imperfection during the direct bonding of two silicon PhCS, some chemical oxide like SiO_2 may be produced in between the two silicon layers with a thickness of 0.6 – 1.2 nm as presented in Ref.[1]. Here we choose the gap thickness of 3 nm to evaluate the influence of the bonding imperfection. We find the proposed structure

and its χ-point BIC have considerable tolerance against the air/SiO_2 gap between the two layers. The radiated polarization states are mostly maintained as shown in Figure S17.

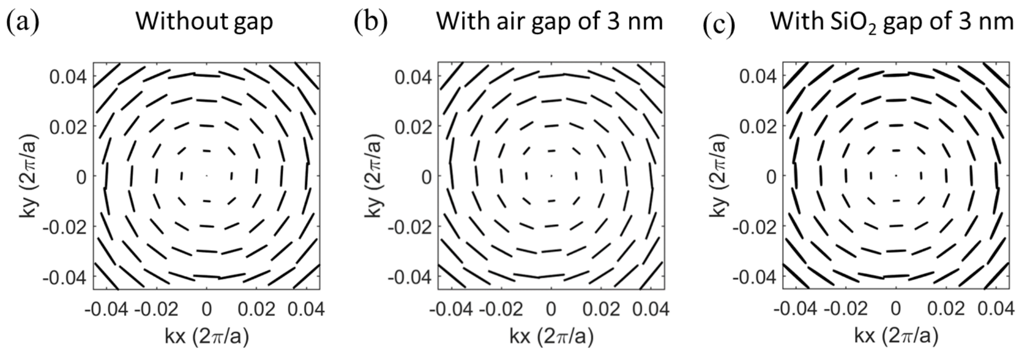

Figure S17. Impact of gap between the two layers with $\alpha_{top} = 36^\circ$. Corresponding polarization field (a) at perfect bonding condition without any gaps, (b) with air gap of 3 nm and (c) with SiO_2 gap of 3 nm.

Supplementary Note 12: Discussion on optical efficiency.

The twisted PhCS with χ-point BIC is made of all-dielectric materials like silicon in the infrared range, and works under the condition with/without other surrounding materials like SiO2, PMMA, index-match liquid that fill the air hole. All these dielectric materials have near-zero material loss (imaginary refractive index). Therefore, if the excitation is considered, the light will be either transmitted through or reflected by the twisted PhCS (no absorption). As we demonstrated in the main text Figure 5 with bi-directional radiation, where both upward and downward radiation have the same and tunable topological distribution around χ-point BIC. Hence the combination of reflected and transmitted channels of polarization field is able to achieve near unity efficiency in theory

References

[1] Shanhui Fan, Wonjoo Suh, and J. D. Joannopoulos. Temporal coupled-mode theory for the Fano resonance in optical resonators. *Journal of the Optical Society of America A*, 20(3):569, 3 2003.

[2] Wonjoo Suh, Zheng Wang, and Shanhui Fan. Temporal coupled-mode theory and the presence of non-orthogonal modes in lossless multimode cavities. *IEEE Journal of Quantum Electronics*, 40(10):1511–1518, 2004.

[3] Chia Wei Hsu, Bo Zhen, Marin Soljačić, and A. Douglas Stone. Polarization state of radiation from a photonic crystal slab. *arXiv:1708.02197*, 8 2017.

[4] Adam Overvig, Nanfang Yu, and Andrea Alù. Chiral Quasi-Bound States in the Continuum. *Physical Review Letters,* 126(7):73001, 2021.

[5] Edward Collett. *Field Guide to Polarization*. SPIE Press, Bellingham, WA, 2005.

[6] Wenzhe Liu, Bo Wang, Yiwen Zhang, Jiajun Wang, Maoxiong Zhao, Fang Guan, Xiaohan Liu, Lei Shi, and Jian Zi. Circularly polarized states spawning from bound states in the continuum. *Physical Review Letters*, 123(11):116104, 2019.

[7] Bo Zhen, Chia Wei Hsu, Ling Lu, A. Douglas Stone, and Marin Soljačić. Topological nature of optical bound states in the continuum. *Physical Review Letters*, 113(25):1–5, 2014.

[8] TaiRong Bai, Qian Li, YiQing Wang, YiFan Chen, Zheng-Da Hu, and Jicheng Wang. Terahertz vortex beam generator based on bound states in the continuum. *Optics Express*, 29(16):25270, 8 2021.

[9] Chia Wei Hsu, Bo Zhen, Jeongwon Lee, Song Liang Chua, Steven G. Johnson, John D. Joannopoulos, and Marin Soljačić. Observation of trapped light within the radiation continuum. *Nature*, 499(7457):188–191, 2013.

[10] Katsuya Tanaka, Dennis Arslan, Stefan Fasold, Michael Steinert, Ju.rgen Sautter, Matthias Falkner, Thomas Pertsch, Manuel Decker, and Isabelle Staude. Chiral bilayer all-dielectric metasurfaces. *ACS Nano*, 14(11):15926–15935, 11 2020.

[11] Giovanni Milione, H. I. Sztul, D. A. Nolan, and R. R. Alfano. Higher-order poincaré sphere, stokes parameters, and the angular momentum of light. *Physical Review Letters*, 107(5), 7 2011.
